# Treatment effect, postoperative complications, and their reasons in juvenile thoracic and lumbar spinal tuberculosis surgery

**DOI:** 10.1186/s13018-015-0300-y

**Published:** 2015-10-01

**Authors:** Qing-Yi He, Jian-Zhong Xu, Qiang Zhou, Fei Luo, Tianyong Hou, Zehua Zhang

**Affiliations:** Department of Orthopedics, Southwest Hospital, Third Military Medical University, No. 30 Gaotanyan Street Shapingba District, Chongqing, 400038 China

**Keywords:** Juvenile, Thoracic and lumbar spinal tuberculosis, Postoperative complications

## Abstract

**Objective:**

Fifty-four juvenile cases under 18 years of age with thoracic and lumbar spinal tuberculosis underwent focus debridement, deformity correction, bone graft fusion, and internal fixation. The treatment effects, complications, and reasons were analyzed retrospectively.

**Material and method:**

There were 54 juvenile cases under 18 years of age with thoracolumbar spinal tuberculosis. The average age was 9.2 years old, and the sample comprised 38 males and 16 females. The disease types included 28 thoracic cases, 17 thoracolumbar cases, and 9 lumbar cases. Nerve function was evaluated with the Frankel classification. Thirty-six cases were performed with focus debridement and deformity correction and were supported with allograft or autograft in mesh and fixed with pedicle screws from a posterior approach. Eight cases underwent a combined anterior and posterior surgical approach. Nine cases underwent osteotomy and deformity correction, and one case received focus debridement. The treatment effects, complications, and bone fusions were tracked for an average of 52 months.

**Results:**

According to the Frankel classification, paralysis was improved from 3 cases of B, 8 cases of C, 18 cases of D, and 25 cases of E preoperatively. This improvement was found in 3 cases of C, 6 cases of D, and 45 cases of E at a final follow-up postoperatively. No nerve dysfunction was aggravated. VAS was improved from 7.8 ± 1.7 preoperatively to 3.2 ± 2.1 at final follow-up postoperatively. ODI was improved from 77.5 ± 17.3 preoperatively to 28.4 ± 15.9 at final follow-up postoperatively. Kyphosis Cobb angle improved from 62.2° ± 3.7° preoperatively to 37° ± 2.4° at final follow-up postoperatively. Both of these are significant improvements, and all bone grafts were fused. Complications related to the operation occurred in 31.5 % (17/54) of cases. Six cases suffered postoperative aggravated kyphosis deformity, eight cases suffered proximal kyphosis deformity, one case suffered pedicle penetration, one case suffered failure of internal devices, and one case suffered recurrence of tuberculosis.

**Conclusion:**

As long as the treatment plan is fully prepared, the surgical option can achieve a satisfactory curative effect in treating juvenile spinal tuberculosis despite some complications.

## Background

In developing countries such as China, India, and Turkey, spinal tuberculosis is one of the primary causes of spinal deformity and paralysis [1]. Regardless of whether an operation is performed or not, chemotherapy is an internal part of the management of spinal tuberculosis. An operation may be performed to drain abscesses, to debride sequestered bone and disc, to decompress the spinal cord, or to stabilize the spine for the prevention or correction of deformity [2]. Spinal tuberculosis (TB) causes neurological complications and gross spinal deformity, which in children increases even with treatment and after achieving healing [3]. Approximately 5 % of patients with spinal tuberculosis will develop a severe kyphotic deformity. When tubercular lesions result in progression of kyphosis to more than 50°, the deformity should be surgically corrected to avoid problems associated with sagittal imbalance [4]. The surgical treatment of spinal tuberculosis, which combined anterior lesion debridement and posterior bone graft fusion and fixation in lumbar and thoracic tuberculosis in one or two stages, has achieved good curative effect [5–8]. For adolescent spinal tuberculosis, analysis of the causes of the complications and related reasons is seldom studied. In this paper, 54 adolescent spinal tuberculosis cases that were treated surgically were studied, and the operation method, curative effect, complications, and related reasons were analyzed.

## Materials and methods

### Physical data

Among the 54 patients studied, there were 38 male cases and 16 female cases. The average age of patients was 9.2 years (range of 2–18 years old, with 32 cases under 10 years of age). The average duration of treatment was 8.7 months (range of 1–28 months).

### Morbidity position

Twenty-eight cases were thoracic spinal tuberculosis, 17 cases targeted the thoracolumbar segment, and nine cases were lumbar spinal tuberculosis. Twenty-two cases suffered two lesions of vertebral bodies, 25 cases had three or more lesions of vertebral bodies, and seven cases suffered the jumping type of vertebral tuberculosis. Thirty-seven cases exhibited a preoperative combined deformity, yielding a deformity rate of 68.5 %, and 29 cases suffered from incomplete paraplegia, according to the Frankel classification. This consisted of three type B cases, eight type C cases, and 18 type D cases. Seven cases had cold abscess fistula. The preoperative kyphosis Cobb angle is 62.2° ± 3.7°.

### Therapeutic method

For the chemotherapy treatment, effective anti-tuberculosis drugs were selected [1]. All patients were given isoniazid, rifampin, and ethambutol for 2 or more weeks. If the effect was positive, the protocol is continued, but if the effect was not favorable, the pyrazinamide is added to form a quadruple chemotherapy drug treatment. Erythrocyte sedimentation rate (ESR), liver function, and kidney function were reviewed once monthly. If there were any cold abscesses, aspiration under CT guidance and a tuberculosis bacterium culture were performed, and the drugs were adjusted according to drug susceptibility results. If there were any fistula or mixed infections, proper antibiotics could also have been used [2]. For supportive treatment, a high protein and high vitamin diet was advocated to strengthen nutrition. If necessary, a small amount of plasma or albumin was given to patients to improve the patients’ conditions [3]. Proper immobilization with a brace was implemented for thoracic and lumbar spine instability lesions. When there was a sign of a cold abscess breaking on its own, computed tomography (CT)-guided repeated aspiration was performed on the abscess, and an anti-tuberculosis drug was injected into the abscess cavity. Pus was sent for tuberculosis bacterium cultures and a drug susceptibility test.

### Operative treatment

#### Operation method

The posterior approach, anterior column tuberculosis debridement, deformity correction by osteotomy, titanium mesh supporting the anterior column with autograft or allograft, and supplementation with posterior pedicle screws were performed in 36 cases. A combined anterior and posterior approach was performed in eight cases, and nine cases of spinal tuberculosis with severe kyphosis deformity were corrected by osteotomy. Simple debridement of tuberculosis lesions was performed in one case. The surgical curative effects, complications, and fusions were observed for an average of 52 months after treatment.

#### Postoperative management

Thoracolumbar tuberculosis patients were immobilized with a brace for at least 3 months and continued receiving chemotherapy with isoniazid, rifampin, and ethambutol for at least 1 year.

#### Statistical method

The Frankel classification, involving vertebral Cobb angles, VAS (visual analogue scale), and ODI (The Oswestry Disability Index) scores at the preoperative stage and at final follow-up, were recorded and analyzed using SPSS statistical software package, with *P* < 0.05 considered significant.

### Ethical approval

This study was conducted in accordance with the declaration of Helsinki. This study was conducted with approval from the Ethics Committee of Southwest Hospital, Third Military Medical University. Written informed consent was obtained from all participants.

## Results

This group comprised 54 cases, and the duration of follow-up was 24–84 months, with an average of 52 months. The age of surgery, types of surgery, preoperative angles, postoperative angles, and angles at final follow-up were summarized in Table 1. Using the preoperative Frankel grade of paralysis, three cases of class B, eight cases of C, 18 cases of D, and 25 cases of E were found. Frankel paralysis of class C occurred in one case, class D was seen in three cases, and class E was observed in 50 cases at final follow-up. No nerve dysfunction was aggravating. VAS from 7.8 ± 1.7 before the operation improved to 3.2 ± 2.1 at the last follow-up. The ODI score of 77.5 ± 17.3 before the operation improved to 28.4 ± 15.9 at the last follow-up. The kyphosis Cobb angles of involved vertebrae improved from 62.2° ± 3.7° preoperative to 37° ± 2.4° at the final follow-up. The above indicators were significantly improved (*P* < 0.05), with all of the cases achieving bone fusion (Table 2).

**Table 1:** Cohort of age of surgery, types of surgery, preoperative angles, postoperative angles, and angles at final follow-up

	Age of surgery	Types of surgery	Preoperative angles (degrees)	Postoperative angles (degrees)	Angles at final follow-up (degrees)
1	5	P	60	35	38
2	5	A + P	65	13	13
3	6	P + O	66	35	38
4	13	P	66	36	36
5	8	P	60	39	39
6	11	P	68	35	35
7	8	P	58	41	43
8	16	A + P	64	37	39
9	10	P	64	36	36
10	12	P + O	70	34	36
11	10	P	68	35	36
12	17	P	65	45	48
13	9	P	59	36	38
14	11	A + P	60	10	10
15	8	P	62	33	37
16	15	P	57	50	53
17	6	P	60	58	62
18	14	P	64	41	42
19	8	P + O	68	35	35
20	18	A + P	57	33	34
21	2	P	64	33	35
22	4	P + O	64	50	53
23	4	P	45	8	8
24	9	P	63	53	54
25	7	P + O	64	35	36
26	7	P	60	50	61
27	8	A + P	65	29	31
28	7	P	63	37	36
29	5	P	60	33	33
30	10	P	65	32	33
31	8	P	65	34	34
32	13	P	58	38	38
33	8	P	46	10	46
34	14	A + P	63	38	41
35	8	P + O	52	35	35
36	12	P	61	49	57
37	8	P	61	37	37
38	9	P	57	36	37
39	11	A + P	68	38	38
40	6	P	70	38	41
41	9	P	59	45	47
42	10	P	60	38	40
43	14	P	60	35	47
44	9	P	62	38	37
45	6	P + O	66	8	8
46	10	P	62	40	40
47	12	P	61	40	40
48	8	P	62	38	38
49	9	A + P	66	38	37
50	6	P	63	38	37
51	7	P	65	35	36
52	10	P	62	36	40
53	8	P + O	67	8	8
54	10	P + O	67	9	9

*P* posterior approach, *A + P* anterior approach + posterior approach, *P + O* osteotomy from posterior approach

**Table 2:** Result summary before and after surgery in Frankel grade, VAS, ODI, and kyphosis Cobb angle

		Preoperative	Postoperative at final follow-up
Frankel grade	A	0	0
	B	3	0
	C	8	1
	D	18	3
	E	25	50
VAS (points)		7.8 ± 1.7	3.2 ± 2.1
ODI (points)		77.5 ± 17.3	28.4 ± 15.9
Kyphosis Cobb (°)		62.2 ± 3.7	37 ± 2.4

*VAS* visual analogue scale, *O* *DI* The Oswestry Disability Index

**Fig 1:**
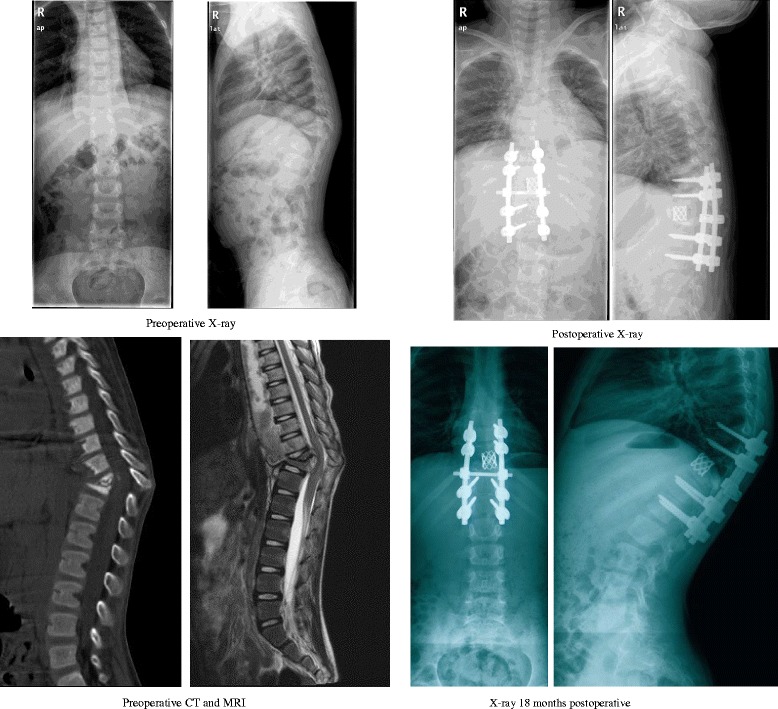
Female, 8 years, T9, T10, and T11 TB with paravertebral cold abscess formation. Preoperative X-ray AP and lateral view, kyphotic Cobb angle of 46°; preoperative two-dimensional sagittal CT, T10, T11 vertebral severe destruction; preoperative MR, spinal cord was compressed obviously in T10 and T11 levels with paravertebral cold abscess formation; postoperative X-ray AP and lateral view, kyphotic Cobb angle of 10°; X-ray AP view 18 months postoperative: T8 vertebral pedicle screw cutting, proximal adjacent kyphosis, Cobb angle 46°

The surgery-related complication rate was 31.5 % (17/54). Six complications were cases of kyphosis deformity aggravated, eight were cases of adjacent segment kyphosis deformity (Fig. 1), one was a case of pedicle cut, one was a case of internal fixation failure, and one case was of tuberculosis recurrence. Demographic and neurological status and surgery-related complications in 17 cases are summarized in Table 3. There were no cerebrospinal fluid leakages, worsening of postoperative neurologic symptoms, drug-induced liver and kidney function damage, optic nerve damage, pressure ulcers, urinary tract infections, or other complications.

**Table 3:** Demographic, TB pathology, neurological status, and surgery-related complication rate in 17 cases

	Age	Sex	Tuberculosis pathology	Neurological injury	Surgery method	Osteotomy	Complication	Outcome
1	7	M	T6–T10	Fankel C	Posterior	Osteotomy	Kyphosis aggravated	Fankel D
2	6	F	T8–T10	None	Posterior	Osteotomy	Kyphosis aggravated	Intact
3	12	F	T9–T10	None	Posterior	Osteotomy	Kyphosis aggravated	Intact
4	8	F	T10–T11	None	Posterior		Pedicle cut	Intact
5	8	F	T11, T12	Fankel D	Posterior		Proximal adjacent kyphosis	Fankel E
6	5	M	T11, T12	Fankel D	Anterior + posterior		Internal fixation broken	Fankel E
7	11	F	T12–L1, L2	None	Anterior + posterior		Proximal adjacent kyphosis	Intact
8	15	F	T9–T12	None	Posterior		Kyphosis aggravated	Intact
9	5	M	T8–T10	None	Posterior		Proximal adjacent kyphosis	Intact
10	14	F	T8, T9	Fankel D	Posterior		Proximal adjacent kyphosis	Fankel E
11	16	M	T10–T12	None	Anterior + posterior		Proximal adjacent kyphosis	Intact
12	18	F	T12–L1	None	Anterior + posterior		TB recurrence	Intact
13	4	F	T10–L1	Fankel C	Posterior	Osteotomy	Kyphosis aggravated	Fankel E
14	17	M	T12–L3	None	Posterior		Kyphosis aggravated	Intact
15	6	M	T9–T12	Fankel D	Posterior		Proximal adjacent kyphosis	Fankel E
16	14	M	T8, T9	None	Posterior		Proximal adjacent kyphosis	Intact
17	11	M	T10, T11	None	Anterior + posterior		Proximal adjacent kyphosis	Intact

## Discussion

Neurologic recovery was rather good after surgery in child and adolescent spine tuberculosis. Bailey and colleagues [9] reported 20 cases of spinal tuberculosis with younger than 11 years after surgical decompression; 85 % of children with nerve dysfunction get fully recovered in 2–36 months. Tuli [10] reported that neural functional recovery rate of spinal tuberculosis was 76–83 %. Moon [11, 12] reported that neural functional recovery rate of spinal tuberculosis was 86–91 %. There were 25 cases (86.2 %) fully recovered; Frankel classification improved one to two grades in 4 cases in our group. The treatment effect was comparable with that of the above report.

Spinal tuberculosis in children and adolescents are more likely to be affected by more segments and kyphosis deformity forming. Fifty-four patients included here involved two or more segments of vertebral body. Twenty-two cases involved 2 vertebral bodies, 25 cases involved 3 or more vertebral bodies, and 7 cases involved jumping-type vertebral tuberculosis. Rajasekaran [13] reported that involvement of segmental spinal tuberculosis in children was 1.9 times that of adults, and the kyphosis deformity in spinal tuberculosis in children and adolescents were more common. They found that the average kyphosis Cobb angle of more than 60° and spine in growth period were more likely to lead to serious spinal deformity in spine tuberculosis. Thirty-seven patients suffered kyphosis deformity among 54 patients (68.5 %) in our group. Spinal tuberculosis in children less than 10 years old, vertebral body loss of more than 1–1.5, pre-treatment deformity angle of greater than 30, and involvement of cervicothoracic or thoracolumbar junction are the other risk factors for deformity progression [14–16]. The average age of our 54 cases was 9.2 years old. Thirteen cases under the age of 10 proceed into kyphosis deformity, which is consistent with the above reports.

Surgical complication rate of spinal tuberculosis in children and adolescents was relative high. Chandra [17] studied the curative effect of 179 cases of spinal tuberculosis surgical complications postoperatively; the incidence of complications was 56.6 %. Using 4 kinds of surgical treatment in 117 cases of thoracic spinal tuberculosis with 2–6 years old for 10 years of follow-up, Schulitz [18] observed that anterior TB lesion debridement and bone graft fusion in 49 cases resulted in aggravated kyphosis deformity with average of 12° Cobb angle. Of 20 patients with posterior approach TB lesion debridement and bone graft fusion, the average kyphosis Cobb angle has not changed; Anterior TB lesion debridement combined with posterior bone graft fusion in 28 cases resulted in reduced average of 7° Cobb angle in kyphosis deformity; Simple anterior TB lesion debridement in 20 case resulted in reduced average of 4° Cobb angle in kyphosis deformity. Combined anterior TB lesion debridement and posterior bone graft fusion can obtain good growth rate and deformity correction. In this group of patients, unlike Schulitz’s report, even if combined anterior and posterior approach surgery was performed in this group of children aged less than 10, 31.5 % of the incidence of kyphosis deformity occurred (17/54). Analysis reasons of 14 cases of patients with aggravated kyphosis deformity and distal junction kyphosis deformity among 17 complicated cases are as follows: one reason is small onset age. Average age of 14 patients is 8.3 years. For children under the age of 10, especially during the age of 2–10, spine is at the peak of growth and development. Studies have found that children less than 7 years old have a higher risk of kyphosis deformity [15]. The second reason is that tuberculosis damages more segments and the TB damage is located in the thoracic segments or thoracolumbar segment. The segments and locations of the destruction in 14 patients were distributed more in thoracic and thoracolumbar segments: 4 cases of T8–T10 (3 segments), 2 cases of T10–L1 (4 segments), 3 cases of T9–T12 (4 segments), 5 cases of T12–L2 (3 segments). The study found that more than three segments of thoracic and thoracolumbar spinal tuberculosis predisposed the bigger rate of kyphosis deformity. The third reason is the influence of operation method. Simple anterior approach debridement and bone graft fusion will result in postoperative aggravated kyphosis deformity.

Possible solution to postoperative kyphosis deformity and adjacent kyphosis deformity was suggested. Non-surgical treatment should be the first choice for too young patients with spine tuberculosis. The widely recognized indications of spinal tuberculosis are the following: progressive neurological deficit, severe spinal deformity, spinal instability leading to pain [19, 20]. With no nerve deficit and no spinal instability of children, especially 2–10-year-old children at the spine growth peak, nutrition support and targeted chemotherapy treatment should be applied to these children as far as possible then close follow-up at least 6–8 years. Therefore, of the surgical treatment for 1000 cases of spinal tuberculosis in 12 years in our hospital, only 54 cases of children with spinal tuberculosis were operated. For Oguz [21], type I, even some type II and III cases, conservative treatment should be pursued.

**Fig 2:**
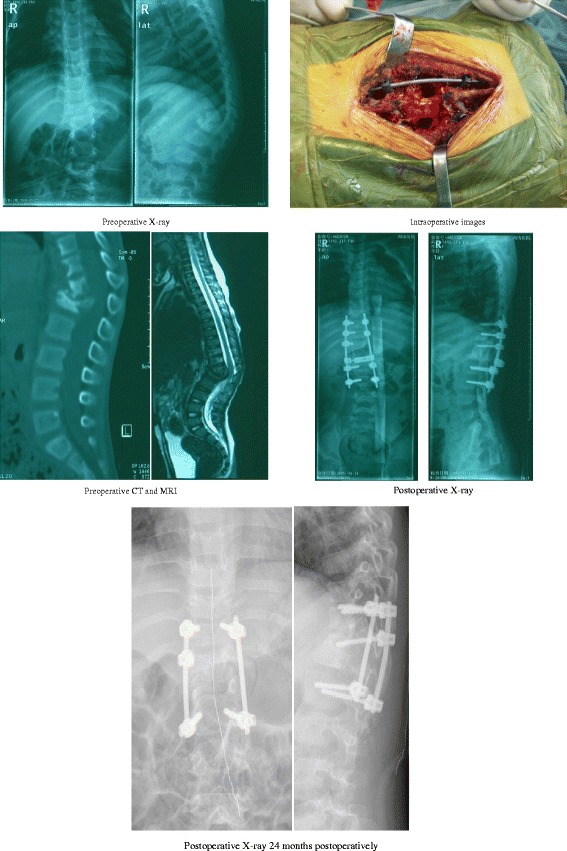
Female, 4 years, T11 and T12, L1 TB with paravertebral cold abscess formation. Preoperative X-ray AP and lateral view, kyphotic Cobb angle of 45°; preoperative two-dimensional sagittal CT, T12 and L1 vertebral severe destruction; preoperative MR, spinal cord was compressed at the T12, L1 level; Intraoperative images, anterior TB lesion debridement, titanium mesh implantation with autologous bone graft + posterior pedicle osteotomy and pedicle screw fixation; postoperative X-ray AP and lateral view, pedicle screws fixation at T10, T11, T12, L2, L3, autogenous bone grafting and fusion range limited between T11, T12 and L2 with kyphotic Cobb angle 8°; postoperative X-ray AP and lateral view 24 months postoperatively, T10, L3 pedicle screws were removed, keeping T11, T12, L2 vertebral pedicle screw fixation; successfully bone fusion range limited between T11, T12, and L2 with kyphotic Cobb angle 8°

Through increasing the number of pedicle screws, increasing anchor fixed point, and fusing more segments, the goal of increasing fixation and orthopedic force could be achieved, thus reducing the possibility of kyphosis deformity, but fusing too many segments will affect the children’s growth and development. In order to solve this dilemma, more segments could be fixed but only tuberculosis-damaged segments were fused. After bone fusion was achieved (6–12 months), the internal fixation in non-fused segments could be removed; this strategy not only increased the intensity of internal fixation and limited the range of bone graft fusion but kept the mobility of the spinal segments of outside lesions as well (Fig. 2).

The authors acknowledge some limitations of this study. As a retrospective study, data collection, treatment, and diagnostic techniques were not standardized and controlled as those in a prospective study. These patients with juvenile thoracic and lumbar spine tuberculosis were carefully selected, which may pose bias to the study to some degree.

### Bullet point summary

- Non-surgical treatment should be the first choice in juvenile spine tuberculosis, especially in patients of less than 10 years old.
- Surgical indications for young children (age <10 years) are the progressive neurological deficit and the aggravating kyphosis according to our study.
- Surgery can achieve satisfactory curative effect in treating juvenile spine tuberculosis in selected cases.
- The major postoperative complications were postoperative proximal kyphosis and aggravated kyphosis deformity in children aged less than 10.

## Conclusion

In selected cases, the surgical option can achieve a satisfactory curative effect in treating juvenile spinal tuberculosis. The main complications of surgery were the postoperative aggravated kyphosis and adjacent kyphosis deformity. Reasons of complications included the patient’s young age, more tuberculosis lesion segments involved, and the selected operation method.
